# Data for the marble-cement paste composites for sustainable construction

**DOI:** 10.1016/j.dib.2019.104528

**Published:** 2019-09-17

**Authors:** Ertug Aydin, Hasan Şahan Arel

**Affiliations:** a Department of Civil Engineering, European University of Lefke, Lefke, TR-10, North Cyprus, Turkey; b Faculty of Architecture, İzmir University, Gürsel Aksel Bulvarı, No. 14, 35350, Üçkuyular, İzmir, Turkey

**Keywords:** Marble powder, Cement, Paste, Composite, Sustainability, Construction, Waste

## Abstract

Waste utilization is crucial for achieving sustainability in the construction industry. This article provides data that demonstrate the possibility of industrial waste utilization in high volumes to enhance cementitious composites. This dataset could help researchers understand the cement paste composites’ behavior in the fresh and hardened state. The data were obtained from the physical, mechanical, and durability tests of laboratory-produced samples. The composites were tested at 7, 28, and 90-days of hardening, according to the American Society for Testing and Materials (ASTM) standards. The obtained values were then evaluated using the DataFit curve fitting (nonlinear regression) and data plotting software. Additionally, two spreadsheets were created to help researchers calculate their weights and calculate the bulk specific gravity, water absorption, and porosity values. The detailed information related to the data described here can be found in “High-volume marble substitution in cement-paste: towards a better sustainability” [1].

**Table:** Specifications Table

Subject	Materials Science (General), Engineering
Specific subject area	Civil and Structural Engineering
Type of data	Table, Image, figure, text file
How data were acquired	Physical, mechanical and durability tests at the age of 7, 28 and 90-days of hardening. DataFit curve fitting (nonlinear regression) and data plotting software were used to analyze the experimental measurements.
Data format	Raw, analyzed
Parameters for data collection	Five different mixture groups composed of high amount of marble powder (60–100% by weight) waste and cement were used to prepare the composites. The water to binder ratio was kept constant at 48%. The composites were tested at 7, 28 and 90-days of hardening.
Description of data collection	Data was obtained from laboratory experiments at the age of 7, 28 and 90-days of hardening by mini slump, water absorption, dry unit weight, porosity, unconfined compressive strength, flexural strength, ultrasonic pulse velocity and weight loss by sodium sulphate tests.
Data source location	TR- 10 Turkey, Lefke, Northern Cyprus
Data accessibility	The all data herein and supplementary files are all available within this article.
Related research article	Aydin, E; Hasan A.Ş, High-volume marble substitution in cement-paste: towards a better sustainability, J. Cleaner Prod. 2019, 237C, 10.1016/j.jclepro.2019.117801

**Table:** 

**Value of the Data** • The dataset in this article can be used to examine the physicomechanical properties of pure marble cement paste composites. • The researchers can design their mix proportioning methods to investigate the durability properties of pure pastes. • The other industrial wastes can be utilized using the same methods here to investigate the microstructure of pure marble cement paste composites. • This dataset provides sustainable building products for the construction sector. • The dataset can be used as an alternative binder for cement production.

## 1 Data

**Fig. 1:**
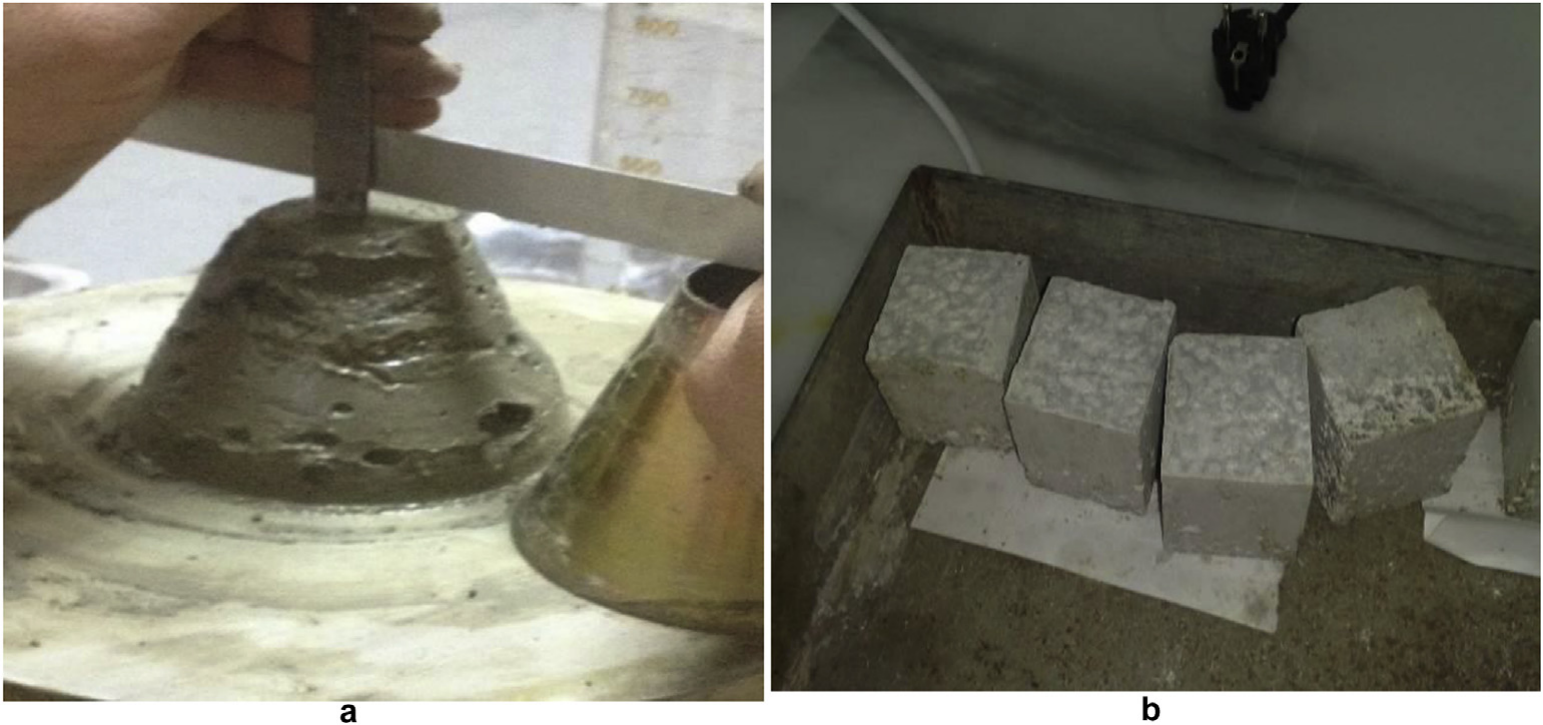
(a) Mini-slump test for workability. (b) Selected samples.

**Fig. 2:**
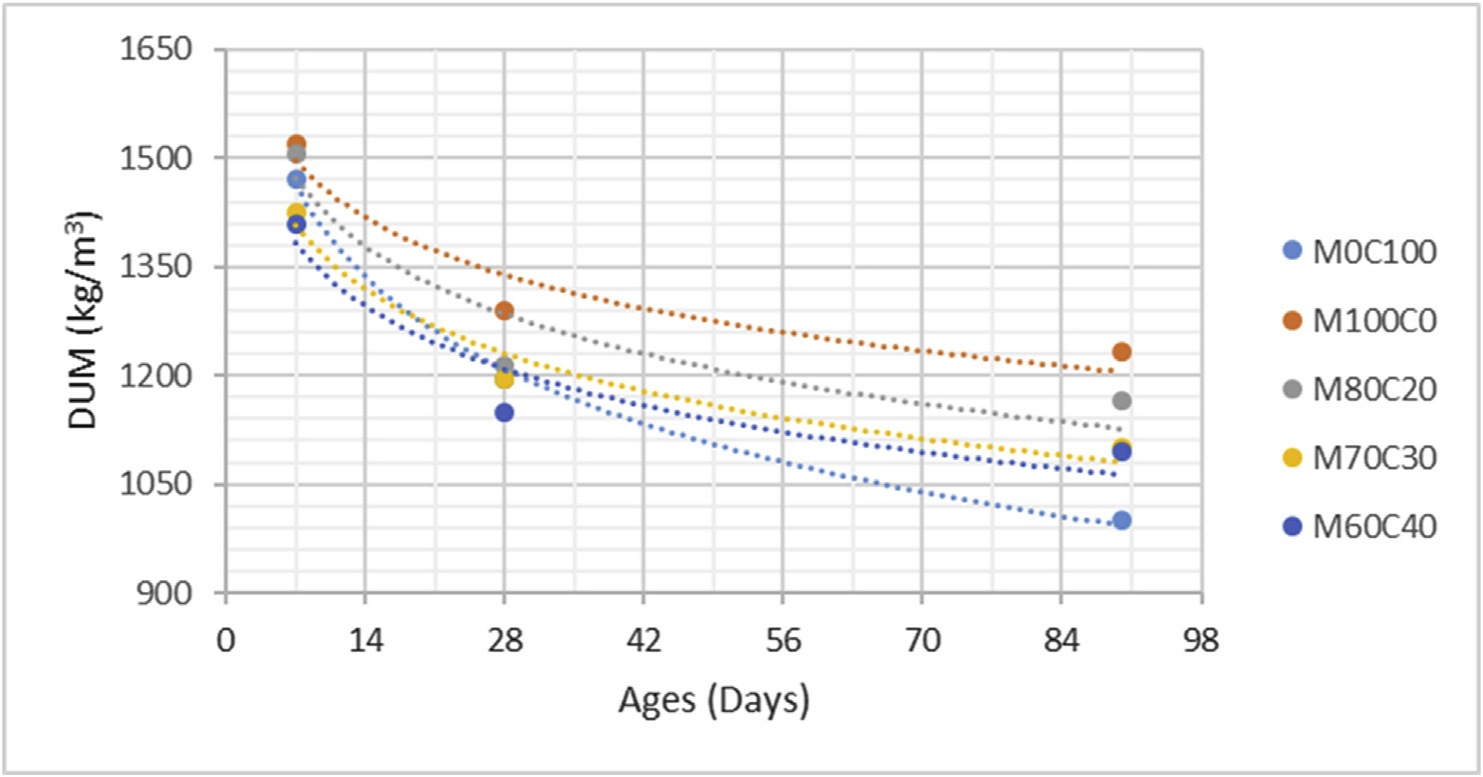
Dry unit mass versus for high-volume marble-cement paste composites at 7–28–90 days of hardening.

**Fig. 3:**
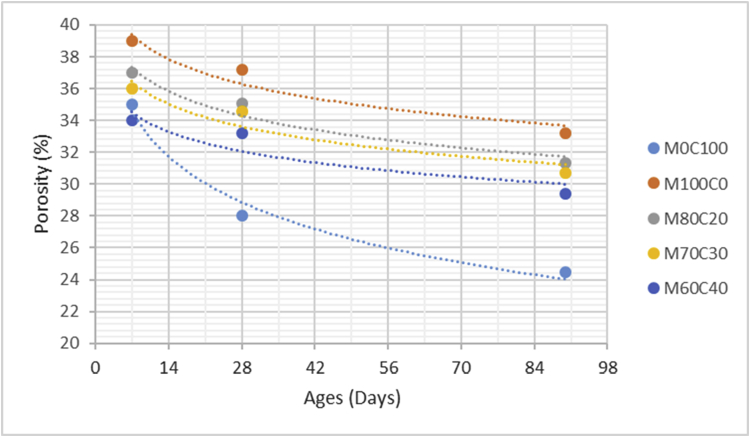
Porosity values for high volume marble-cement paste composites at 7–28–90 days of hardening.

**Fig. 4:**
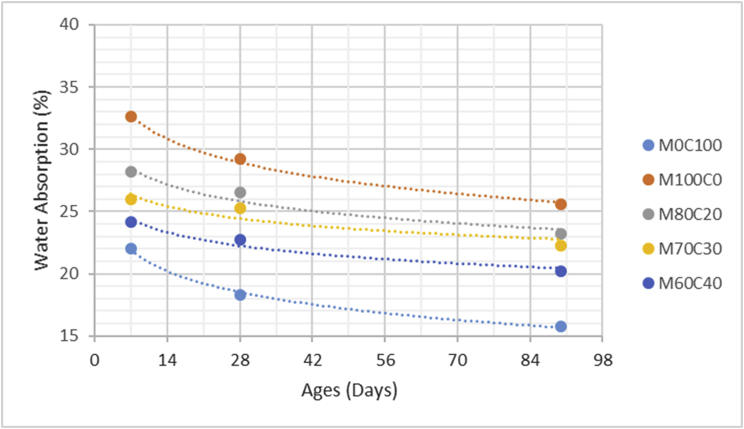
Water Absorption values for high volume marble-cement paste composites at 7–28–90 days of hardening.

**Fig. 5:**
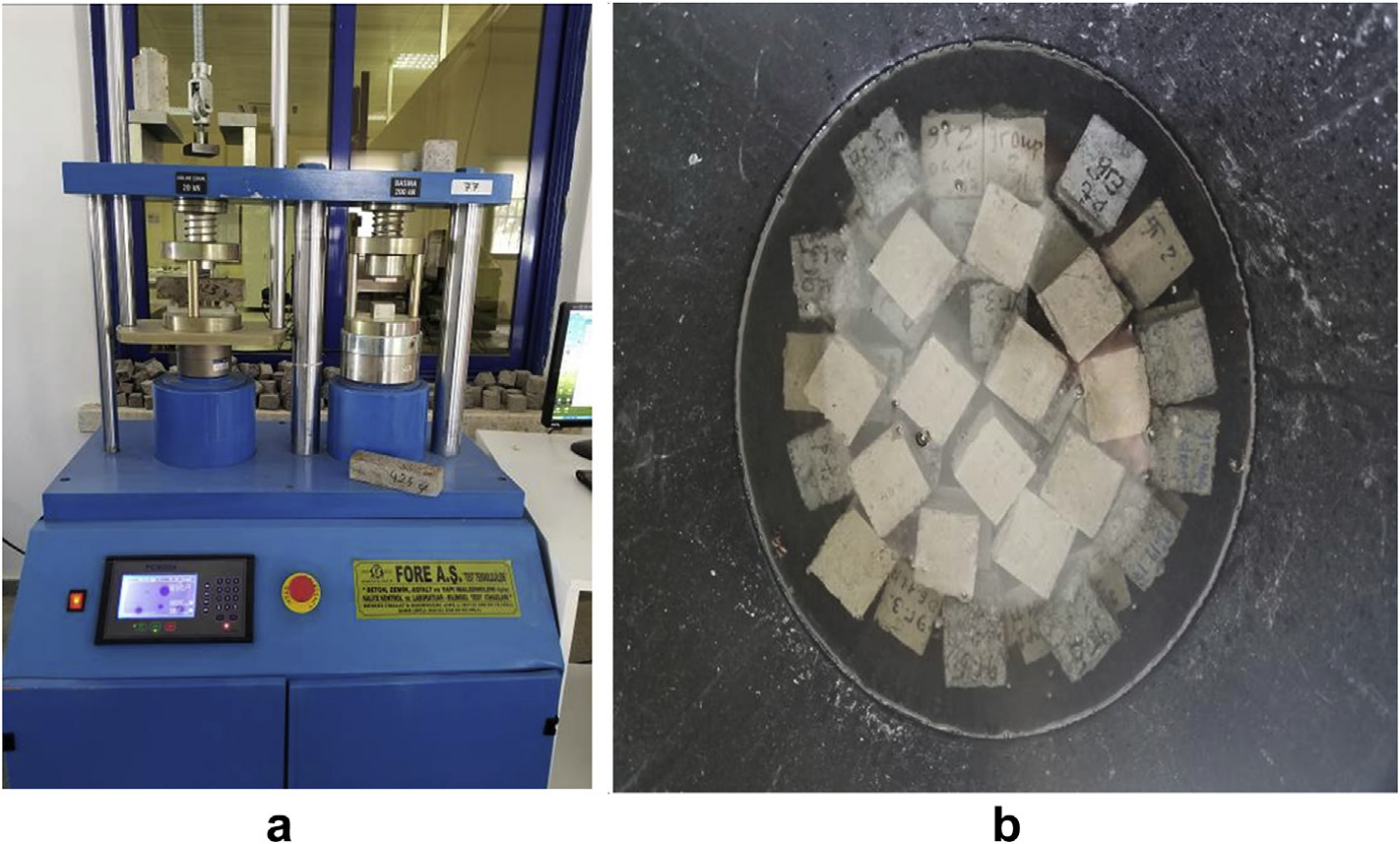
(a) Strength testing set-up. (b) Sulfate testing of selected samples.

**Fig. 6:**
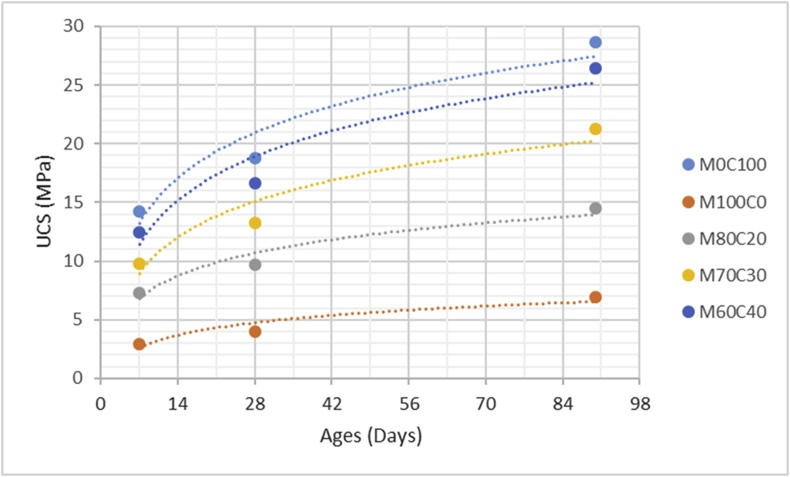
Compressive strength values for high volume marble-cement paste composites at 7–28–90 days of hardening.

**Fig. 7:**
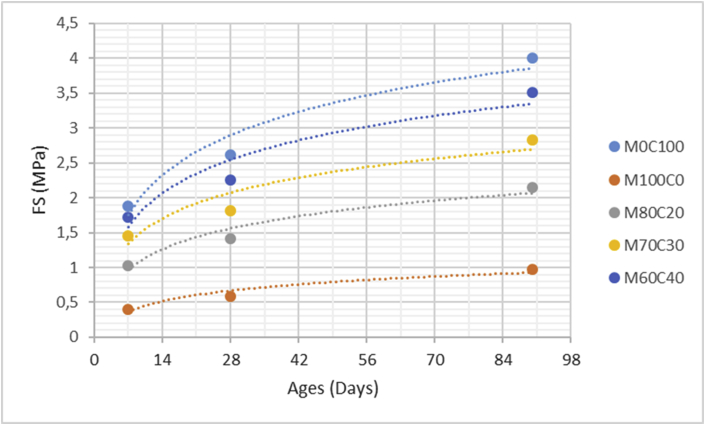
Flexural strength values for high volume marble-cement paste composites at 7–28–90 days of hardening.

**Fig. 8:**
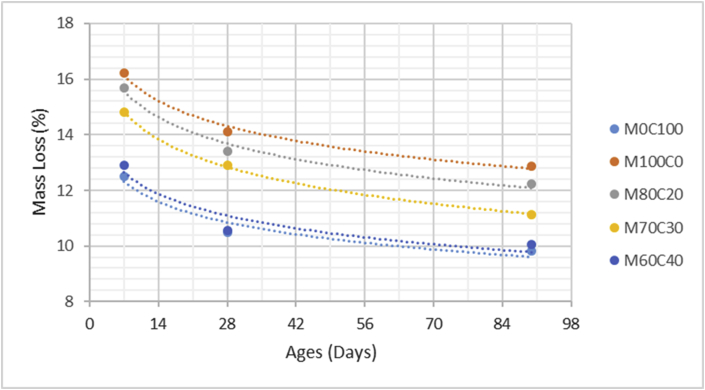
Mass loss by sodium sulfate for high volume marble-cement paste composites at 7–28–90 days of hardening.

**Fig. 9:**
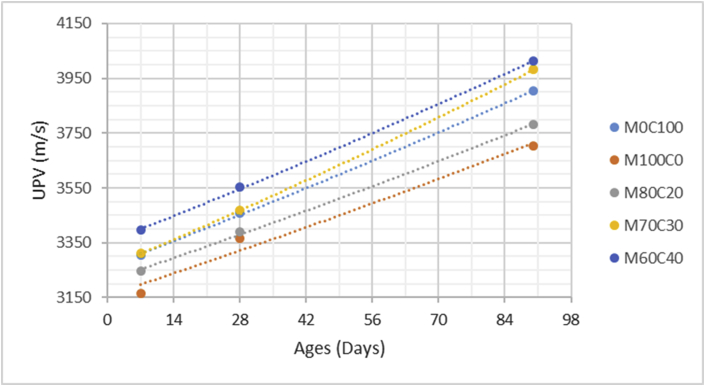
Ultrasonic pulse value for high volume marble-cement paste composites at 7–28–90 days of hardening.

The data comprised fresh and hardened properties of the marble-cement paste composites. Pure cement paste composites were prepared on a laboratory scale. The composites were then evaluated based on the mini-slump test, as shown in Fig. 1a for workability. The produced marble cement paste samples are shown in Fig. 1b. This data article presents dry unit weight (DUW), porosity, water absorption, unconfined compressive strength (UCS), flexural strength (FS), weight loss by sulphate solution and ultrasonic pulse velocity (UPV). The data were divided into four categories based on physical, mechanical, durability and quality tests. Table 1 shows all data measured during this research. Fig. 1a shows the mini-slump test for workability (first category). Mini slump can help researchers identify the consistency of the mixtures. The method is easy to apply, especially for pastes and mortars. The fifty cubic millimetres of produced samples during this research are shown in Fig. 1b. In all Figures, M denotes marble dust and C denotes the cement. The numbers represent the mass percentages used in mixture proportioning. Fig. 2, shows dry unit weight values for all mixture groups. The second category of data presented in Fig. 3, Fig. 4 represents durability performance at 7, 28, and 90 days of hardening. Porosity and water absorption measurements shown in Fig. 3, Fig. 4 were chosen as durability parameters to classify the produced samples. Fig. 5a shows the compressive and flexural strength test set-up used in this research. Fig. 5b shows the selected samples immersed in a sulphate solution. In the third category tests, UCS and FS test measurements are presented in Fig. 6, Fig. 7. The fourth category concerns the quality evaluation of produced samples presented in Fig. 8, Fig. 9 based on the 7, 28, and 90 days of hardening. Additionally, two excel worksheets are provided to help researchers calculate weights and calculate the specific gravity, water absorption, and porosity values. Items highlighted in yellow should be entered during laboratory studies. More detailed information can be found both in supplementary Excel files accompanying this article and in Refs. [1], [8].

**Table 1:** All measured data at 7, 28 and 90 days of hardening periods.

Analysis	Curing time (days)	M0C100	M100C0	M80C20	M70C30	M60C40
DUW (kg/m^3^)	7	1470	1520	1505	1425	1410
	28	1195	1290	1215	1195	1150
	90	1000	1232	1165	1100	1095
Porosity (%)	7	35	39	37	36	34
	28	28	37,2	35,1	34,6	33,2
	90	24,5	33,2	31,3	30,7	29,4
WA (%)	7	22	32,6	28,2	26	24,2
	28	18,3	29,2	26,5	25,3	22,7
	90	15,8	25,6	23,2	22,3	20,2
UCS (MPa)	7	14,23	2,95	7,26	9,82	12,47
	28	18,75	4,03	9,72	13,25	16,64
	90	28,63	6,95	14,48	21,24	26,45
FS (MPa)	7	1,88	0,4	1,03	1,45	1,72
	28	2,62	0,59	1,41	1,82	2,25
	90	4,01	0,97	2,15	2,83	3,51
Mass loss (%)	7	12,5	16,21	15,7	14,8	12,9
	28	10,48	14,12	13,41	12,9	10,55
	90	9,81	12,88	12,23	11,12	10,07
UPV (m/s)	7	3305	3165	3245	3310	3395
	28	3458	3367	3390	3467	3552
	90	3903	3702	3780	3981	4012

## 2 Experimental design, materials, and methods

The data presented herein were obtained by blending a high amount of marble with cement. No treatment was applied to marble powder that was used directly in mixtures proportioning to achieve better sustainability. First, the actual water content of the marble powder was determined and subsequently, the amount of water in the composites was adjusted for every mixture groups. The laboratory-produced composites were designed as pure paste, i.e., no fine or coarse aggregate was available in any of the mixture proportioning. The water to binder ratio for all groups was kept constant at 48%. This value was optimized based on previous studies [2], [3], [4]. Five different mixture groups were prepared. The reference group was designated as M0C100 (marble weight was considered as zero and weight of cement was considered as 100%). Similarly, the mixture group M80C20 comprised 80% marble powder and 20% cement. Composites were cast in 50 mm cubic formwork and 40 mm × 40 mm × 160 mm prismatic molds. The prepared samples were tested at 7, 28, and 90-days of hardening. Composites were evaluated based on the ACI report [5] and ASTM standards [6], [7]. The detailed mix proportions, experimental setup, and information can be found in Ref. [1], and datasets can be found in Ref. [8].
